# Exploring the Experiences of Community-Dwelling Older Adults on Using Wearable Monitoring Devices With Regular Support From Community Health Workers, Nurses, and Social Workers: Qualitative Descriptive Study

**DOI:** 10.2196/49403

**Published:** 2024-08-07

**Authors:** Arkers Kwan Ching Wong, Jonathan Bayuo, Jing Jing Su, Karen Kit Sum Chow, Siu Man Wong, Bonnie Po Wong, Athena Yin Lam Lee, Frances Kam Yuet Wong

**Affiliations:** 1 School of Nursing The Hong Kong Polytechnic University Kowloon China (Hong Kong); 2 Hong Kong Lutheran Social Service Kowloon China (Hong Kong); 3 Department of Health The Government of the Hong Kong Special Administrative Region Hong Kong Island China (Hong Kong)

**Keywords:** community-dwelling older adults, focus group, wearable monitoring devices, mobile phone

## Abstract

**Background:**

The use of wearable monitoring devices (WMDs), such as smartwatches, is advancing support and care for community-dwelling older adults across the globe. Despite existing evidence of the importance of WMDs in preventing problems and promoting health, significant concerns remain about the decline in use after a period of time, which warrant an understanding of how older adults experience the devices.

**Objective:**

This study aims to explore and describe the experiences of community-dwelling older adults after receiving our interventional program, which included the use of a smartwatch with support from a community health workers, nurses, and social workers, including the challenges that they experienced while using the device, the perceived benefits, and strategies to promote their sustained use of the device.

**Methods:**

We used a qualitative descriptive approach in this study. Older adults who had taken part in an interventional study involving the use of smartwatches and who were receiving regular health and social support were invited to participate in focus group discussions at the end of the trial. Purposive sampling was used to recruit potential participants. Older adults who agreed to participate were assigned to focus groups based on their community. The focus group discussions were facilitated and moderated by 2 members of the research team. All discussions were recorded and transcribed verbatim. We used the constant comparison analytical approach to analyze the focus group data.

**Results:**

A total of 22 participants assigned to 6 focus groups participated in the study. The experiences of community-dwelling older adults emerged as (1) challenges associated with the use of WMDs, (2) the perceived benefits of using the WMDs, and (3) strategies to promote the use of WMDs. In addition, the findings also demonstrate a hierarchical pattern of health-seeking behaviors by older adults: seeking assistance first from older adult volunteers, then from social workers, and finally from nurses.

**Conclusions:**

Ongoing use of the WMDs is potentially possible, but it is important to ensure the availability of technical support, maintain active professional follow-ups by nurses and social workers, and include older adult volunteers to support other older adults in such programs.

## Introduction

### Background

Technological advancements have facilitated the self-management of chronic diseases among community-dwelling older adults. Wearable monitoring devices (WMDs), such as smartwatches, are among the common technological tools that assist older adults with health monitoring, physical and cognitive training, medication reminders, and fall prevention [1,2]. The literature shows that WMDs are effective at reducing the risk of developing cardiovascular diseases [3], increasing the physical activity levels [4], and improving the quality of life [5] of older adults. However, despite the benefits and high adoption rate of these wearable devices, there is a lack of studies demonstrating the adherence rate of older adults in maintaining consistent use of WMDs [6-8]. A survey with a sample of >4000 Canadian adults revealed that 33% of the participants did not use WMDs to monitor their health on a regular basis [9]. Similarly, another survey conducted in Australia reported an abandonment rate of 29% for WMDs, without specifying the population [10]. Physical disability, a lack of knowledge about the functions of wearable devices, and technological anxiety were summarized as notable reasons for poor adherence to these devices among older adults [11-13].

Self-determination theory has highlighted that long-term behavioral change is determined by one’s intrinsic motivation, which is defined as one’s action driven by the enjoyment and interest in the activity itself and is affected by 3 factors: competence, autonomy, and relatedness [14]. When an individual has a sense of competence and autonomy in adopting a new behavior and has someone who is socially and psychologically connected (relatedness) to support the behavior, they are more likely to adhere to, and maintain, the behavior over the long term. Recent studies have focused on providing training sessions to help older adults familiarize themselves with the functions of WMDs and enhance their competence and autonomy. However, the results showed no difference in adherence between the participants who received the training sessions and those who did not [15,16]. Older adults have expressed in a qualitative study that 1 preintervention training session is not sufficient to enhance their knowledge of WMDs or resolve their technological anxiety [17]. It was suggested that nursing or peer support, with the simultaneous provision of social support, might be necessary throughout the health program to increase the intrinsic motivation of older adults to adopt WMDs [15]. However, there have been limited studies on the offering of nursing or peer support for older adults in the use of WMDs. In the study by Farivar et al [11], nursing feedback was provided to older adults when their real-time step counts, which were displayed on the WMDs, were unsatisfactory. The program was found to be feasible and acceptable to older adults, but it encountered challenges such as infrequent updates of the WMDs and low engagement and retention rates. Another study, which designed a similar program that provided nursing support to older adults when abnormal vital signs were detected in the WMDs, demonstrated a high dropout rate of 21% and short-term adherence to the WMDs [18]. Recent studies emphasize the importance of implementing a clear nursing service model, such as a case management model, that encompasses problem identification, goal setting, and regular follow-up. This model aims to enhance the intrinsic motivation of older adults to consistently use new technologies, such as mobile health apps and WMDs [7,19], rather than relying solely on providing training sessions to them or intervening only when abnormalities in vital signs are detected through WMDs by the older adults.

### Objectives

Because of the perception that they might be causing trouble to others, older adults tended not to actively seek help from health care professionals and peers even when they faced technical problems or when they did not comprehend the medical jargon displayed on the device [11,13]. They were also concerned about their health data being transferred from WMDs to health care professionals but not receiving regular feedback [20]. In view of this, this study was to have a nurse case manager (NCM) work with the older adults to identify factors that could facilitate or hinder their use of a smartwatch and recommended that the older adults use those features of the smartwatch that are linked with their health and social problems, provided suggestions on the duration and frequency of the use of the smartwatch, and provided instructions on how to use these features in their daily routines during the 3-month intervention period. Older adults had the autonomy to adjust or modify their own schedules to ensure that they could use the features of the smartwatch efficiently and effectively. The NCM also encouraged the family members or primary caregivers of the older adults to participate and provide feedback and support. This paper describes the perceptions and experiences of community-dwelling older adults after receiving our interventional program. More specifically, we explored the challenges that they experienced during their use of the WMD, the benefits of using the WMD, and suggestions on how to promote their sustained use of the WMD. The results may provide useful insights for developing a program that can promote the continued adoption of WMDs and, in turn, improve the long-term benefits of the WMDs on health self-management among older adults.

## Methods

### Study Design

A qualitative descriptive design was adopted for this study [21]. This approach is not associated with any philosophical or theoretical orientation but draws on naturalistic inquiry to understand and describe how people experience a phenomenon [21]. The qualitative descriptive study is the method of choice when straight descriptions of phenomena are desired, which made it appropriate for this study. This study is reported according to the SRQR (Standards for Reporting Qualitative Research) checklist [22].

### Setting and Participants

This study was conducted between June 2022 and March 2023 in collaboration with 5 community centers run by a local nongovernmental organization in Hong Kong. Using a purposive sampling approach, the members of these community centers who were interested in this program were screened and recruited into this study if they (1) were aged ≥60 years [23], (2) owned a smartphone, (3) were able to communicate in Cantonese or Mandarin, and (4) had internet access. They were excluded if they (1) had been diagnosed with cognitive impairment, (2) were bedbound, (3) owned a smartwatch, and (4) were involved in other studies using WMDs.

### Recruitment Process

Staff working at the collaborating community centers invited their members to join the program using Facebook Live. Those who were interested provided their name to the staff, and trained research assistants screened them via telephone. Eligible members were invited to meet the research assistants at the community centers to receive an explanation of the details of the study and to give their written consent to take part in it. All participants received a health monitoring package that included a smartwatch with an alarm setting, a prepaid SIM card, a blood pressure monitor, and a pulse oximeter.

### Interventional Program

Before the program, a 1-hour web-based training session and a practical test were delivered to all participants to explain the basic operation of the WMD. The number of a telephone manned during office hours by staff of the community center was provided to participants to call should they face any technical problems during use.

The participants were provided with a package that included a WMD (ProVista Care smartwatch), a prepaid SIM card, a blood pressure monitor, and a pulse oximeter. ProVista Care was selected as the WMD for this study due to its validated performance, affordability, and comparable functionality to other similar devices. These functions encompass fall detection; location and activity tracking; blood pressure, pulse, and oxygen saturation monitoring; medication and appointment reminders; and calls to preset numbers and SOS calls. This selection enhances the applicability of the study’s findings to real-world implementation. Data collected from ProVista Care can be synchronized and transferred to the server via the ProVista Care app installed on participants’ personal smartphones. The WMD was designed to be worn on the wrist, securely fastened with an elastic band. Participants were instructed to wear the WMD as frequently and for as long as possible throughout the study duration.

Ten trained community health workers (CHWs), NCMs, and social workers were the interventionists in this 3-month program. The participants in the intervention group received a home visit by a CHW and the NCM in the first month and biweekly telephone calls by the CHW from the 3rd to the 12th weeks. In the first home visit, using the Omaha System, the NCM explored the features of the smartwatch that each participant might find beneficial. The Omaha System is a comprehensive assessment-intervention-evaluation instrument for community-based practice [24]. There were 21 health and social problems listed in the Omaha System that were relevant to the features of the smartwatch used in this study; for example, the feature of fall detection in the smartwatch might help participants with musculoskeletal problems or lower limb weakness. The NCM empowered the participants to set goals and action plans in the first meeting, while the CHWs followed up, recalling the goals and action plans with the participants and, in subsequent telephone calls, motivating the participants to regularly use the smartwatch. The NCM also monitored the vital signs of the participants that were automatically uploaded by the smartwatches at the backend. When abnormal vital signs were detected by the smartwatch, the NCM would call the participants via telephone and provide appropriate interventions, such as a referral to a social worker, based on the validated protocols.

### Data Collection

A total of 6 in-depth focus group discussions with 22 participants were conducted at the end of this program. In-depth focus group interviews are conducted to evaluate participants’ experiences after an intervention through group interactions [25]. For studies using focus group discussions, it has been suggested that groups ranging from 2 to 40 may be adequate to attain data saturation depending on the phenomenon under investigation [25]. Thus, 6 groups were considered adequate for this study to attain data saturation. All discussions were conducted with a guide developed by the research team. The focus group interviews were conducted in Cantonese and each session lasted for 25 to 65 minutes. All interviews were audio recorded with the consent of the participants. Interview transcripts were written up by members of the research team. To ensure the consistency of coding and interpretation of data, an audit trail was conducted, and all discrepancies were resolved through discussion and consensus.

### Data Analysis

All data collected from the focus group discussions were analyzed inductively using the approach to constant comparison analysis formulated by Maykut and Morehouse [26], who proposed a 4-step approach to the constant comparison of focus group data: inductive categorization, refinement of categories, exploring relationships across the categories, and integration of data [26]. In inductive categorization, AKCW and JB read and reread the interview transcripts in both English (JB) and Cantonese (AKCW) to identify recurring concepts independently. Next, overlapping concepts across the groups were categorized and combined by the 2 independent coders (AKCW and JB) to formulate provisional codes. In the second stage, that is, refining the categories of codes, the provisional list of codes was concurrently examined alongside a review of the interview transcripts. The process of categorization was undertaken through discussion with the wider research group to attain consensus. Subsequently, similar codes were grouped to formulate categories for each group. The emerging categories were then concurrently compared across the groups, with recurring categories further refined and grouped. For the third stage, we further refined the categories by grouping them under a distinct umbrella. Categories with common elements were grouped to make broader groups or emerging themes. With these themes, we worked through each group and the associated categories to attain a complete understanding and create patterns of meaning from the data.

### Methodological Rigor and Trustworthiness

The trustworthiness of this study was evaluated according to four criteria: (1) credibility, (2) dependability, (3) confirmability, and (4) transferability [27]. To enhance credibility and dependability, the summarized results were sent to those participants who had agreed to check them for further clarification and to give feedback on the researchers’ interpretation. Audit trails and peer debriefings were also conducted during the analysis of data to ensure the consistency of the interpreted data to achieve confirmability. A thick description was ensured in reporting the study to enhance transferability. To attain analytical rigor, we ensured that analyses were undertaken in both Cantonese and English and compared to ensure consistency. The authors responsible for this section were fluent in Cantonese and English. The iterative approach to analysis also ensured consistent coding, with an audit trail on the decisions on coding and categorization. In addition, the constant comparison approach ensured that our focus was not only on individual-level analyses but also on analyses within and across the groups.

### Ethical Considerations

This study was conducted under the principles of the Declaration of Helsinki and approved by the ethics committee of the Hong Kong Polytechnic University (HSEARS20220429001). All eligible participants were given the right to refuse participation and the right to withdraw from the interview at any time. Written informed consent was collected from all participants. To protect the participants’ privacy, all data collected from this program were kept confidential and anonymized and were only accessible to the members of the research team.

## Results

### Sociodemographic Data

A total of 22 community-dwelling older adults were involved in 6 focus group discussions. Of these, 5 (23%) were male, and 17 (77%) were female, with ages ranging from 62 to 78 years. Only 1 (5%) participant had experience in using a smartwatch before inclusion in the study. A total of 17 (77%) had a primary level of education, and 5 (23%) had a secondary level of education or higher. The clinical characteristics of the participants have been reported in a previous study [19].

### Emerging Themes and Categories

#### Overview

Three themes and 7 categories emerged from the focus group data, as shown in Textbox 1.

**Emerging themes and categories.**

**Challenges associated with the use of the wearable monitoring device (WMD)**

- Individual-related challenges
- System-related challenges

**Perceived benefits of using the WMD**

- Self-monitoring and health promotion
- Convenience

**Strategies to promote use of the WMD**

- Availability of technical support
- Ongoing follow-up professional support
- Peer and family support

#### Emerging Theme 1: Challenges Associated With the Use of the WMD

This theme describes challenges and concerns that affected the participants’ use of the smartwatch. The emerging categories were (1) individual-related challenges and (2) system-related or technical challenges.

#### Individual-Related Challenges

Participants across all groups emphasized that they were slow in learning to use the WMD and required a great deal of face-to-face instruction to be fully oriented to the device and its functionalities before being able to use it effectively. This issue particularly resonated with those who were using such a device for the first time. Some of the older adults either could not understand the instructions or needed more time to assimilate them. It took weeks to months for the older adults to become familiar with the device:

This is my first time wearing a smartwatch. When you wear the watch for the first time, you will definitely not know how to use all the functions. So, I wanted to ask everyone if they have experienced this situation before.

Participant 1

Well...at first, it was difficult to use. But after using it for a while, it was basically okay, and we can use it on our own.... Hmm yes, actually, if someone teaches you face-to-face, you can learn it clearly first.

Participant 15

How much time? I think three to four months to learn to use it well.

Participant 20

For me, at first, it took a long time to use it. Sometimes, I just could not get it to work. But after a while, it became much better. For example, measuring blood oxygen and blood pressure readings became much easier for me with time.

Participant 4

The first time I tried, I did not know how to turn on the device or turn it off. It was difficult at first.

Participant 7

The participants also highlighted the issue of forgetfulness, which affected how well they used the device. They noted that with their increasing age, forgetfulness was a common occurrence. Some of the participants mentioned forgetting how to operate the device and the functions available during the initial period of use, although over time they were able to become better at using the device consistently:

I’m so dumb sometimes that I forget what I am doing. I sometimes cannot even figure out how to wrap a scarf around my head, not to mention how to use the watch.

Participant 13

I am already in my late years. If you even ask me what I ate yesterday, I cannot remember.

Participant 2

#### System-Related or Technical Challenges

System-related or technical challenges were encountered across all groups. The size of the WMD was considered an issue. Participants described the WMD as big, which made it difficult to wear regularly. Occasionally, the size of the watch was considered a source of ridicule. Despite the potential for being ridiculed, some of the older adults noted that they were more concerned about the functionality and capacity of the watch than its size. In addition, the smooth, glass surface of the watch’s touchscreen became slippery and unresponsive when used by the older adults in cold weather, creating usability issues:

The watch can measure blood pressure and blood oxygen levels. Your watch looks much better and looks great. Our watches are big, like big turtles, and sometimes people make fun of it.

Participant 9

You can see that your watch is smaller than ours. Our watch is bigger, and it obstructs a lot. But even if it’s still bulky and unattractive, I think we can still wear it because it will help us.

Participant 20

It feels really troublesome to use during cold weather. There is no problem in hot weather.

Participant 7

The power capacity of the WMD presented a significant challenge for the participants. Participants wanted to use the WMD, but the need for frequent charging made it rather inconvenient to do so. In some cases when they wanted to use it when going out, they noticed that the WMD was low on battery. Coupled with the previously mentioned issue of forgetfulness, this meant that they missed the opportunity to charge it before going out. The participants also reported that the need to frequently charge the WMD prevented them from using it for a longer period throughout the day. This issue deterred some of the older adults from using the WMD altogether on some occasions:

Hmmm, if you know everything, the main problem with the watch is the frequent charging. That battery needs to be charged frequently—like every day. If you don’t charge it, it will just run down fast. Yes, it is so fast and when there is no electricity, things will become difficult. The need to charge is too frequent for us.

Participant 5

Oh, so you realize that the battery is down when you wear it and then you must put it back to charge for a while. Yes, that’s right. It is very troublesome to do this every day before going out. The battery runs out quickly all the time.

Participant 9

Another technical issue that was identified was the fact that some of the participants felt that the WMD had several functions they did not know how to use. Interestingly, other older adults still struggled to navigate through even the few functions that they were taught to use, and they occasionally experienced digital fatigue after constant use:

There may be some functions we cannot use. The watch seems to have many functions, but we do not know them all and also don’t know how to use them all.

Participant 16

But, I realized there are so many functions on that watch that we cannot use them all. Also, some functions that were possible to use before, people found it annoying to continue to use them. That is why we do not use them frequently anymore.

Participant 9

All participants were enthusiastic about the ability of the device to count their steps as they walked about. However, the older adults mentioned that the device gave them incorrect step counts. In 1 group, the participants mentioned that the step count function also did not display correctly. Occasionally, they used their mobile phones to obtain correct step counts. In addition to this challenge, some of the participants reported occasional challenges with uploading or transmitting data on their vital parameters:

The pedometer was malfunctioning and gave incorrect figures. When you count how many steps you take yourself and then check the watch, it doesn’t match at all. The watch and the phone both have incorrect counts all the time.

Participant 8

It shows only a few steps, even though I walked quite a lot. Yes, our watches cannot measure many steps. My phone shows 10,000 steps, but my watch shows 2000 or 3000 steps. To be honest, the watch is not accurate when it comes to the step count. The step count displayed on my phone is not the same as the one on my phone. Yes, that is how it is. There are some differences, yes.

Participant 1

Actually the step count is important, but it is not accurate at all. I often check it myself. Usually, I check how many steps I have taken, especially since I sit in an office for most of the day. But it is not correct when I check the watch and the phone.

Participant 16

I tell him about my blood pressure on that day. I tell him about my blood pressure and how many steps I took that day. Sometimes, the watch cannot display the values correctly.

Participant 9

Some of the participants also found the device to be extremely sensitive, which occasionally caused discomfort because the alarm went off immediately when it sensed a slight movement:

But the watch is too sensitive. Sometimes when I move my hands or feet, it shakes and triggers the alarm. And then it keeps telling me how long it has been and what to do.

Participant 5

#### Emerging Theme 2: Perceived Benefits of Using the WMD

Regardless of the notable challenges, participants highlighted the benefits of using the WMD. These were (1) self-monitoring and health promotion and (2) convenience.

##### Self-Monitoring and Health Promotion

Participants across all groups stated that the WMDs offered them an opportunity to self-monitor some vital parameters, such as blood pressure and oxygen saturation levels. The older adults found this feature to be particularly helpful because it helped them to record their parameters, track them, and share them with health care professionals and to ascertain whether they were maintaining a good health status. Indeed, the use of the device boosted the confidence of older adults across all groups in their ability to actively participate in self-management, particularly because the NCM actively followed up to enable them to attain their health goals:

Um, measure blood pressure and blood oxygen levels at the same time. Well, we know now. We know our blood pressure and blood oxygen levels. It helps us to maintain our health by making us aware of the condition of our body and whether it is normal or not.... At night, I have a blood pressure machine and I can measure my blood pressure every night.

Participant 2

Also, it gives a different perspective on managing your health with more information available to you. For instance, I know how much I walked today.

Participant 8

Yes, definitely. Using the watch gave me a lot of confidence. I wear it at home and when I go out. The nurse also reminded me to walk a certain amount of time every day, and even though I forget, I still try my best to walk more. The most important thing is to try and walk more.

Participant 4

And at home, I don’t know how high or low the blood sugar is. If I know, I can control it by myself at home. If it is high, I will eat less. It is good to be clear about the blood sugar. For the nurses, it would be helpful if they could find my place and remind me of something regularly.

Participant 14

The best function would be to be able to monitor your health and detect any potential illnesses.

Participant 19

The participants expressed a desire for more regular follow-ups by the nurses and an option to monitor their blood glucose levels in addition to blood pressure and blood oxygen levels:

I just think it would have been helpful if the device can also help you to monitor your blood sugar levels just like it helps to monitor blood pressure and blood oxygen levels.

Participant 5

Oh, she sometimes follows up on us with home visits and phone interviews. Yes, but what about the rest of the time? If the nurse does not contact you, you won’t actively look for her, right? Besides, the nurse does not come to the center every day. The nurse is also busy with her work, so where would she have the time? So, in some instances, if you are not feeling better, you go and see a doctor.

Participant 13

Although the step count feature of the WMD was described as inaccurate, the participants felt that it was still helpful to know how many steps they had taken because that motivated them to go out more often rather than stay at home. Being able to compare their step counts with others gave them a sense of accomplishment, especially if they found them to be higher than those of their colleagues:

But I don’t really care so much about how many steps I take in a day. However, it can still calculate something for you. For example, if the doctor tells you how many steps you need to take in a day, the watch can help you to keep track of it. Maybe we don’t really need it because our phones can also count the steps.

Participant 2

I take so many steps every day. Many people can vouch for me. I am the best here; I take so many steps. After finishing my chores at home, I come down and do some healthy dancing, and walk around the center. According to them, I am the best.

Participant 14

Because you can show off to others, like the person you are exercising with, and say, look I have burned this much fat, right?

Participant 9

Participants also mentioned that the device helped them to not only record their vital parameters but also to view these records regularly. Regarding the promotion of health, the participants noted that the device helped them to participate in regular exercises and to build the confidence they need to meet health-related goals:

So, wearing a watch can make you want to do more exercise, right? Because when you wear a watch, you want to see how many steps you have taken, which makes you want to move more.

Participant 6

##### Convenience

For participants across the groups in this study, the WMD offered a sense of convenience in being able to monitor their vital parameters, record the values, track them, and share them with the nurse if required. The notion of convenience was also noted to be related to the ease with which the older adults navigated the device to inform their self-management strategies. In addition, that they did not have to be in a hospital to assess these basic vital parameters was something the older adults considered very convenient. In fact, they could monitor the basic vital parameters from the comfort of their home and even when moving about in the community:

With the watch, there is a guide, and I am afraid to be lazy about moving around and not walking around. But when I think about the watch, I have the confidence to do it. In the past, I just sat at home all day, talking on the mobile phone about how many thousands of steps I have to walk, and now I just go out and do it myself.

Participant 10

In addition, the aspect of being able to reach out and interact with a nurse or having a nurse follow up on an older adult whose parameters were outside the normal range was considered to be convenient. This may be related to the fact that the participants felt that they were not only using the device but also being professionally supported by a nurse:

Yes, if the nurse thinks the blood oxygen is low, she will remind you to do it before and again. Then if something happens to you, you will know to see a doctor.

Participant 5

At least, the blood pressure can be seen by the nurse. And the blood oxygen levels can be seen with a press of the finger. However, the step count is not accurate.

Participant 15

The social aspect of the watch, such as being able to take pictures and share these with families and friends, was considered helpful and made life more convenient for the older adults. In other words, it added a bit of fun to using the device:

I discovered a new function or new feature. It is completely possible to use the watch to take a photo and share. Yes, so it is so much more convenient.

Participant 3

It is best if there is nothing wrong with it. The best thing to do is to take a photo of that watch and the stick together after we finish the test, and it will be the most accurate. It is comfortable and makes life more convenient, I think.

Participant 5

Another source of convenience was noted to be related to the fact that the wearable devices afforded older adults or their families a unique opportunity to track their whereabouts. The older adults found this feature particularly helpful because they considered themselves to be forgetful on occasion, and this feature helped them to retrace their steps to their original location or helped others to know where they were:

The best feature of the device is the tracking. Some people have a poor memory, or they may not be able to find their way home. In that case, their family members can locate them using the tracking feature.

Participant 21

Mr Choi once tracked us. I got lost and could not find my way home. I got scared and started sweating. Mr Choi tracked my watch and found me at the Che Kung Temple.

Participant 2

This is where technology has advanced. The most useful thing is when someone is lost. If he wears the watch, you can find him and track where he has been. Then you can find him using the tracking function.

Participant 14

Sometimes when I go somewhere far, I don’t know where I am, and I cannot see clearly due to my glaucoma. One time, I had to go to the other side for the lunar new year, but I took the wrong bus and did not know where I was. Luckily, I was able to use the watch to track my way.

Participant 6

#### Emerging Theme 3: Strategies to Promote Use of the WMD

This emerging theme discusses approaches observed in the data that highlight strategies to sustain continual use of the device. The following categories were captured: (1) availability of technical support, (2) ongoing follow-up professional support, and (3) peer and family support.

##### Availability of Technical Support

The plethora of technical issues emerging from the use of the device warrants ongoing availability of technical support. This great need was mentioned by participants in all groups and was particularly felt when the device developed a fault or broke down, or the participants needed more assistance in navigating through the functions:

The watch broke down and we did not know how to fix it. Someone at the center said he knew how to turn it back on. We tried for a while, but it still did not work. So, I said forget it. I did not wear it. I only wore it for ten days before, and just for measuring blood pressure at home.

Participant 14

Although some of the participants sought assistance from the social workers, most older adults hesitated to disturb the personnel and therefore avoided seeking assistance altogether, regardless of the technical challenges that they were facing:

So, it is changed. Actually, you also changed and regarded it as a planned situation, and I did not dare to worry the nurse or the supervisor. So, if there is a problem with the watch, I must handle it on my own.

Participant 10

In addition, the participants mentioned that they needed more technical support to access other functions on the watch because they found it difficult to perform this task:

And I don’t understand why so many functions need to be locked, except the panic button. I wondered if there was help for us to unlock these functions on the watch.

Participant 7

We tried to figure it out but could not do it and we needed lots of help. In the end, it suddenly made a sound, and we could not figure out how long it had been, it just happened.

Participant 16

There are too many things to handle. If you suddenly introduce ten functions for us to use, how can we remember them? You are not teaching a class, you won’t be able to remember them either.

Participant 11

##### Ongoing Follow-Up Professional Support

Although the device was helpful in various ways, the older adults still preferred to have nurses follow up actively with them. For the participants across all groups, this form of support was generally limited, and they wished that they had interacted more with the nurses to be able to interpret the values that they obtained and to seek more health-related information. Perhaps the nurse support centered on following up on older adults with abnormal readings. Thus, those who maintained readings within normal ranges had limited contact with the nurses. The participants also felt that the limited support that they received from the nurses might have affected how well they met their health-related goals:

They [the nurses] do a good job when they call or visit you. With the watch, you set a goal with the nurse, which motivates you to do more. But they are not always there. It is helpful if they can find my place so that they can remind me of something I don’t know.

Participant 19

Aside from ongoing professional support from nurses to keep the participants motivated in meeting their health-related goals, support from social workers is equally important to promote their continued use of the devices. Social workers played critical roles in promoting the use of the device by offering troubleshooting support, helping the participants to navigate through the device, and offering encouragement. In fact, it seemed as though the older adults who participated in the study trusted the social workers more than they did the nurses and were willing to always seek assistance from them. The older adults seem to have built a strong relationship with the social workers, which made it easier for them to seek assistance from them if required:

They do help us a lot and encourage us. Whenever there is a problem, we always look for him to help us out. He is the most reliable. He is very responsible, and he is always willing to help us.

Participant 5

I did not even know how to turn off my phone. He said to turn off my phone, do it this way. He really taught us a lot of things.

Participant 10

##### Peer and Family Support

Peer support from the CHWs also emerged as a critical factor to sustain the ongoing use of the WMD. These older adult volunteers or older adult ambassadors often offered encouragement to the participants to continue to use the device, record their values, and work toward meeting their health-related goals. Participants across the groups highlighted that it was occasionally difficult to gain access to a nurse; thus, the older adult volunteers or older adult ambassadors became the first point of call for assistance before reaching out to the social workers:

It is not so easy to find or see a nurse on some days. The volunteers have done this before, so we can reach out to them. There are days when you will forget to write the values, and they will remind you to do so.

Participant 16

Aside from peer support, family support was also observed to be helpful in encouraging the older adults to use the WMD as required. Thus, older adults who resided alone with limited or no family support found it difficult to monitor and continually use the device to promote health:

They said that I fall frequently and have fallen several times before. I must be careful now that I am getting older. If anything happens to me, it would be troublesome because I live alone.

FG2

## Discussion

### Principal Findings

Emerging technologies such as wearable devices are advancing care and support for older adults in communities across the globe. Despite the plethora of literature highlighting the importance of wearable devices, significant concerns remain about the decline in use after a period of time. The world’s aging population is booming, but only a limited amount of work has been done to unearth the experiences of older adults regarding the use of wearable devices. This critical gap informed this study, which was part of a large trial program. The findings bring to the fore the challenges experienced by older adults regarding the use of wearable devices, which were identified as individual- and system-related challenges. The findings of the study further highlight the perceived benefits of the devices, particularly in the areas of self-monitoring, health promotion, and convenience. In addition, the study identified a hierarchical pattern of health-seeking behaviors of older adults when using the devices. Put together, the findings highlight that ongoing use of the devices is possible, although there is a critical need to ensure the availability of technical support and ongoing active professional follow-up by the health care team (notably nurses and social workers) and to include older adult volunteers to support other older adults in such programs.

Previous studies have uncovered various technical issues associated with using wearable devices. In a recent study, the authors identified interoperability, battery issues, the bulky nature of the device, a lack of personalization, and a lack of support as key issues that affected the use of the device [28]. Similar to this finding, our study also noted similar technical issues that affected the use of the devices. By contrast, however, it was noted in our study that regardless of these issues, older adults were willing to continue using the device because they believed that doing so was to their benefit. Despite this, we observed that issues related to individuals can also affect the use of wearable devices among older adults. For most of the older adults included in this study, this was the first time they had to use a wearable device, and they needed more time to become acquainted with it. Although issues such as forgetfulness may be considered part of the aging process, these findings suggest that aside from intensive training on how to use the device, there is still a need for ongoing technical support to boost its use. In addition, instruction manuals need to be more user-friendly and easily comprehensible for older adults. Comprehensibility is essential; we observed in this study that the user manuals were unclear, which may have had an impact on how well the participants made use of the WMD.

The inclusion of professional and peer support in this study is particularly noteworthy. An existing study showed that it might be necessary to provide nursing or peer support throughout the duration of the health program to increase the intrinsic motivation of older adults to adapt to WMDs and to provide social support at the same time [17]. In our study, which combined both professional and peer support, we observed that older adults did not want to disturb the nurses. Rather, they felt more comfortable consulting the older adult volunteers first, before reaching out to the social workers and, last of all, to the nurses if necessary. This hierarchical pattern of health-seeking behaviors may be an indication that older adults viewed the older adult volunteers as peers sharing similar experiences and conditions, which made it easier to relate to them than to the professionals. Nurses were perceived by older adults to be busy professionals. Thus, the participants would rather resort to seeking support from social workers, although they wished they had more interactions with the nurses. Put together, the findings suggest that nurses may need to take an active role in reaching out to older adults and being available when needed, regardless of whether they are using the wearable device. The concept of peer support also needs to be promoted further by engaging other older adults as volunteers to support older adults who are transitioning to using wearable devices. A recent study has shown that peer-to-peer support for community-dwelling older adults has the potential to not only promote adherence to therapeutic regimens but also to improve their quality of life, which warrants further exploration [29].

Furthermore, we observed that the ongoing availability of technical support and family support is also essential to promoting the use of wearable devices. It is possible that technical support may be available but unknown to the older adults or that they may not want to disturb others. Thus, older adults need to be encouraged to seek help if needed and should know where to obtain this help. Regarding family support, it remains a major cornerstone of support for older adults [30]. The absence of this form of critical support may lead to loneliness, which can exacerbate health issues and interfere with therapeutic regimens, including the use of wearable devices [31]. Undoubtedly, expanding on the notion of family support is beyond the scope of this study, but it is possible to recommend that older adults with limited or no family support need to be identified and appropriate strategies devised to assist them.

Moreover, we identified both individual- and system-related issues that can adversely impact the use of WMDs. Individual-related factors such as slow learning patterns and forgetfulness were highlighted by the participants as impacting how they initially navigated the WMD. Undoubtedly, aging is not a disease, although it can be associated with forgetfulness, which can impact activities of daily living [32]. Forgetfulness coupled with the slow learning patterns emphasize the need for continuous, gradual education to enable older adults to use WMDs effectively [33]. System-related challenges such as the size of the WMD and its limited power capacity are concerns that need to be addressed in subsequent design studies. In addition, concerns regarding the WMD generating incorrect readings also emerged as a system-related challenge. Previous studies have reported that a common problem with wearable devices is the likelihood of experiencing automatic loss of synchronization, making it difficult or impossible to update data or resulting in an incorrect report [34,35]. Although loss of synchronization was not examined in this study, it may have potentially contributed to the incorrect readings observed by the older adults in this study.

### Strengths and Limitations

The strength of this study lies in the use of a rigorous approach to collecting and analyzing data with a focus on individual, within-group, and across-group variations to attain a thick description of what it means to experience the use of a wearable device. This strength notwithstanding, some limitations are noteworthy. First, the experiences of the participants are related to the use of a particular wearable device with distinct features. Thus, the findings may not necessarily be transferable to all wearable devices, although they may offer a useful resource on how older adults are likely to experience using the devices. Second, the study was undertaken in a region with distinct sociocultural features. The findings should therefore be interpreted taking these unique features into consideration. In addition, the nature of the program was such that the older adults were required to have some technological abilities. Thus, the findings may not be transferable to older adults who find it difficult to use technological applications.

### Conclusions

Emerging technologies, such as wearable devices, for supporting community-dwelling older adults warrant more work on how users are experiencing these devices. The findings from this study bring to the fore the barriers and benefits of wearable devices and offer insight into strategies that can be considered to improve use. Because of issues that might emerge, it may be helpful to consider the availability of ongoing technical support, professional follow-up support, peer support, and family support. In fact, a personalized approach is needed to promote the use of wearable devices among older adults.

## Abbreviations

CHW: community health worker
NCM: nurse case manager
SRQR: Standards for Reporting Qualitative Research
WMD: wearable monitoring device

## Data Availability

The data sets generated and analyzed during this study are available from the corresponding author on reasonable request.
